# Supplementary figures and images for: Multi-omics analysis and functional validation of CHEK1 as an independent prognostic biomarker in Pancreatic cancer
Source: PLoS One. 2026 Jan 21;21(1):e0340878. doi: 10.1371/journal.pone.0340878 (PMC12822972; doi:10.1371/journal.pone.0340878)

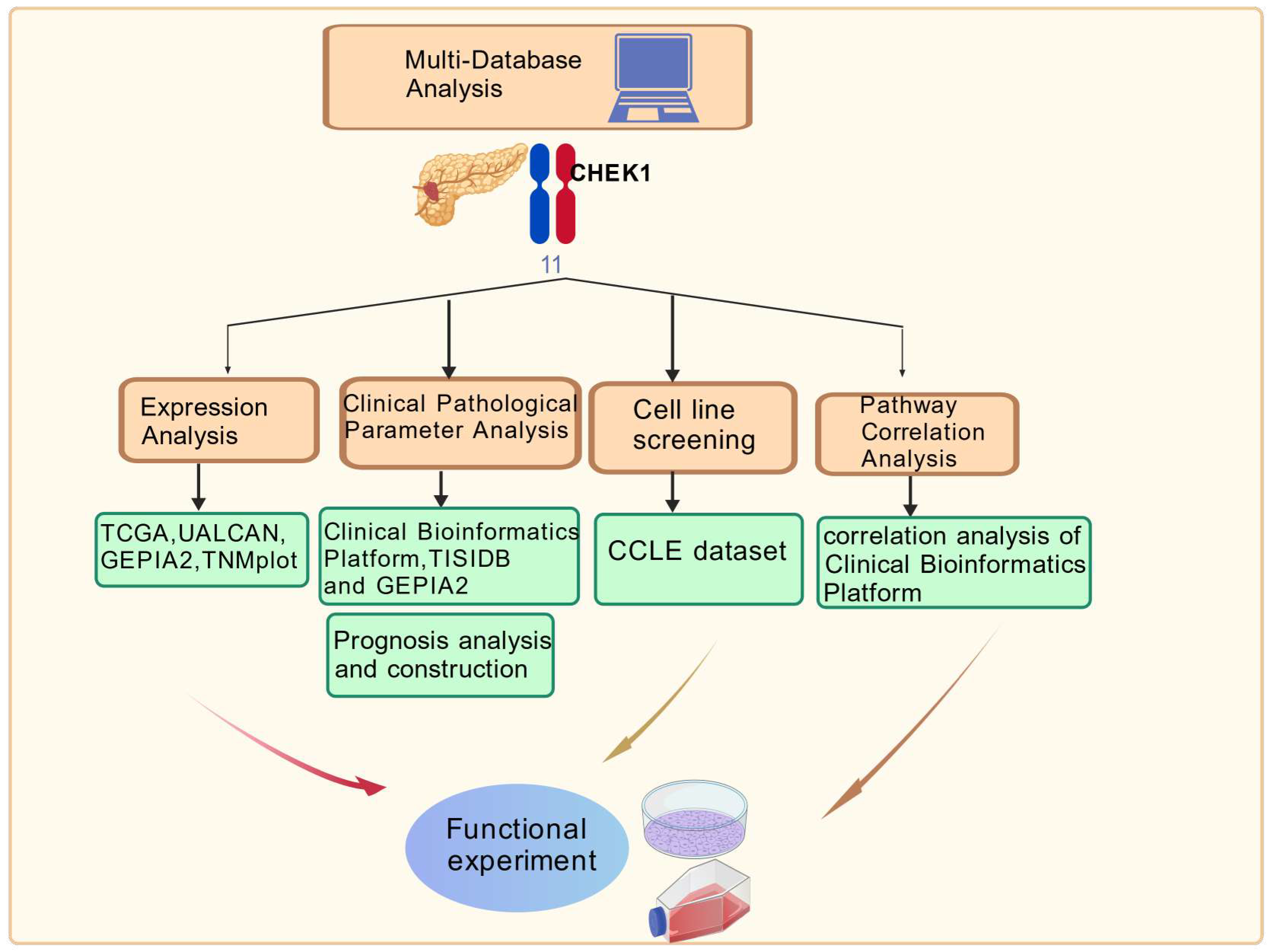

Supplement: S1 Fig — (TIF) [file pone.0340878.s001.tif]

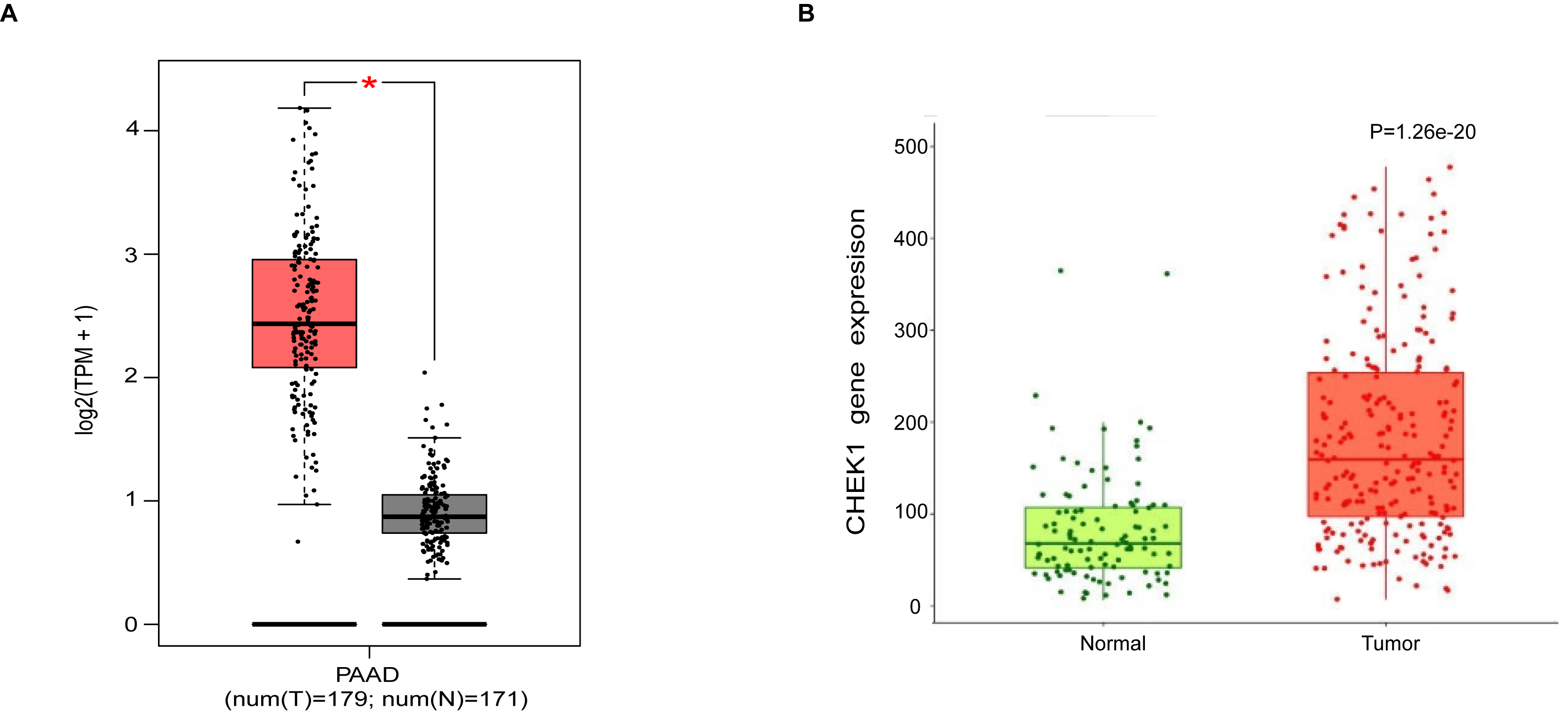

Supplement: S2 Fig — (A) Analyze CHEK1 mRNA expression in pancreatic cancer and normal tissues using the GEPIA 2.0 website. (B) Analyze CHEK1 expression in pancreatic cancer tissue versus normal tissue using RNA sequencing data via the TNMplot online platform. (TIF) [file pone.0340878.s002.tif]

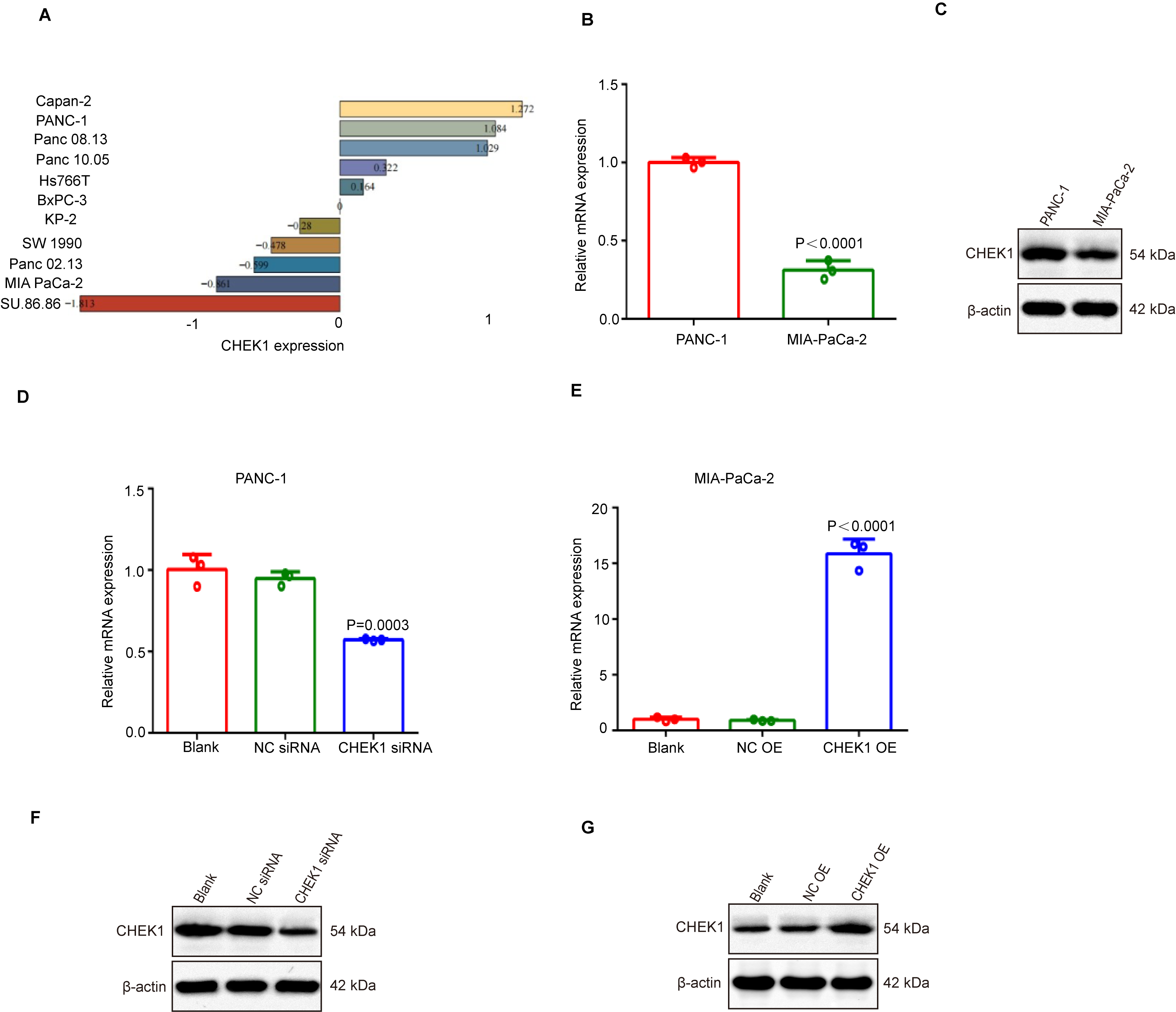

Supplement: S3 Fig — (A) The bar chart illustrates the distribution of CHEK1 expression across various cell lines. In this chart, the horizontal axis denotes the gene expression status, while the vertical axis corresponds to the different cell lines. The height and color of the bars indicate the magnitude of gene expression, with the median value serving as the reference point for division. (B) Analysis of CHEK1 mRNA expression in PANC-1 and MIA PaCa-2 pancreatic cancer cell lines by quantitative real-time PCR (qRT-PCR). (C) Analysis of CHEK1 protein expression in PANC-1 and MIA PaCa-2 pancreatic cancer cell lines by Western blotting. (D) Validation of CHEK1 gene mRNA knockdown in the PANC-1 pancreatic cancer cell line by qRT-PCR. (E) Validation of the overexpression of CHEK1 gene mRNA levels in the pancreatic cancer cell line MIA PaCa-2 by qRT-PCR. (F) Validation of knockdown expression of the CHEK1 gene at the protein level in the PANC-1 pancreatic cancer cell line by Western blotting. (G) Validation of CHEK1 gene overexpression at the protein level in the MIA PaCa-2 pancreatic cancer cell line by Western blotting. (TIF) [file pone.0340878.s003.tif]
