# Supplementary material for: Multi-omics analysis and functional validation of CHEK1 as an independent prognostic biomarker in Pancreatic cancer
Source: PLoS One. 2026 Jan 21;21(1):e0340878. doi: 10.1371/journal.pone.0340878 (PMC12822972; doi:10.1371/journal.pone.0340878)

Fig 4A

**PANC-1 Groups**	Clone number			Cloning efficiency		
Blank	93	87	101	18.60%	17.40%	20.20%
NC siRNA	92	82	89	18.40%	16.40%	17.80%
CHEK1 siRNA	51	45	44	10.20%	9.00%	8.80%

Fig 4B

**MIA-paca-2 Groups**	Clone number			Cloning efficiency		
Blank	86	69	71	17.20%	13.80%	14.20%
NC OE	90	85	65	18.00%	17.00%	13.00%
CHEK1 OE	119	130	116	23.80%	26.00%	23.20%

Fig 4C

**PANC-1 Groups**	Proliferation number	Total Cell Number	Proliferation Rate (%)
Blank	68	235	113.33%
	53	232	89.48%
	49	192	99.96%
NC siRNA	51	188	106.25%
	48	220	85.45%
	49	190	101.01%
CHEK1 siRNA	42	256	64.26%
	33	197	65.61%
	32	176	71.21%

Fig 4D

**MIA paca-2 Groups**	Proliferation number	Total Cell Number	Proliferation Rate（%）
Blank	36	141	100.00%
	41	211	76.11%
	32	155	80.86%
NC OE	30	167	70.36%
	34	134	99.38%
	26	125	81.47%
CHEK1 OE	70	224	122.40%
	73	201	142.25%
	77	202	149.30%

Fig 5A

**PANC-1 Groups**	Freq G1	Freq S	Freq G2
Blank	60.15	30.62	9.07
	61.85	28.24	9.64
	61.91	27.84	10.11
NC siRNA	59.95	28.83	10.93
	58.57	28.68	12.3
	61.07	26.19	12.39
CHEK1 siRNA	75.18	14.17	10.47
	74.11	13.95	11.79
	73.31	15.65	10.95

Fig 5B

**MIA-paca-2 Groups**	Freq G1	Freq S	Freq G2
Blank	72.63	19.44	7.79
	74.8	15.71	9.5
	75.71	15.54	8.73
NC OE	72.2	14.89	12.59
	71.44	16.94	10.71
	72.08	15.29	12.43
CHEK1 OE	66.62	24.56	8.64
	66.38	26.19	7.33
	67.91	23.97	7.96

Fig 6A

PANC-1 Groups	Migratory cell number			% of control		
Blank	217	230	204	106.55%	112.93%	100.16%
NC siRNA	220	201	238	108.02%	98.69%	116.86%
CHEK1 siRNA	87	106	100	42.72%	52.05%	49.10%

Fig 6B

MIA-paca-2 Groups	Migratory cell number			% of control		
Blank	224	190	197	109.98%	93.29%	96.73%
NC OE	235	196	194	115.38%	96.24%	95.25%
CHEK1 OE	385	423	357	189.03%	207.69%	175.29%

Fig 6C

PANC-1 Groups	Relative E-cadherin Expression	Relative E-cadherin Expression	Relative E-cadherin Expression	Relative N-cadherin Expression	Relative N-cadherin Expression	Relative N-cadherin Expression	Relative Vimentin Expression	Relative Vimentin Expression	Relative Vimentin Expression
Blank	0.97105251	1.045443879	0.983503611	0.983870481	1.046385281	0.969744238	0.994673767	1.041912636	0.963413597
NC siRNA	0.946355071	1.002317726	0.88110198	1.011880822	1.070671219	1.099688265	1.052616713	0.999687168	1.030474148
CHEK1 siRNA	1.459334808	1.469459429	1.549675063	0.604375016	0.615333485	0.649535085	0.799555464	0.749907323	0.819934266

Fig 6D

MIA-paca-2 Groups	Relative E-cadherin Expression	Relative E-cadherin Expression	Relative E-cadherin Expression	Relative N-cadherin Expression	Relative N-cadherin Expression	Relative N-cadherin Expression	Relative Vimentin Expression	Relative Vimentin Expression	Relative Vimentin Expression
Blank	0.972005226	1.049756861	0.978237913	0.936406232	1.001698204	1.061895564	0.950684467	1.007855397	1.041460136
NC OE	0.949167847	0.956031896	0.989773734	0.96222352	0.951417498	1.049318615	0.901917843	1.016049969	0.971768057
CHEK1 OE	0.47226543	0.4425942	0.499373844	1.570261514	1.646411671	1.70869904	1.406084107	1.336973231	1.322249364

S3 B

	PANC-1	MIA-PACA-2
2-ΔΔCT	0.968	0.254729424
2-ΔΔCT	1.030778188	0.306594387
2-ΔΔCT	1.002627621	0.373914817

	PANC-1	MIA-paca-2
mean	1.000334717	0.311746209
SD	0.031652272	0.05975948

S3D

	Blank	NC siRNA	CHEK1 siRNA
2-ΔΔCT	0.900	0.98	0.57
2-ΔΔCT	1.078	0.96	0.57
2-ΔΔCT	1.031	0.90	0.58

	Blank	NC siRNA	CHEK1 siRNA
mean	1.00287767	0.948	0.572
SD	0.091766744	0.041	0.006

S3E

	Blank	NC OE	CHEK1 OE
2-ΔΔCT	1.016	0.989	14.349
2-ΔΔCT	0.828	0.850	16.732
2-ΔΔCT	1.189	0.861	16.492

	Blank	NC OE	CHEK1 OE
mean	1.010930222	0.900094927	15.857715
SD	0.180620938	0.077398319	1.31251816

Original WB data

Fig 6

C

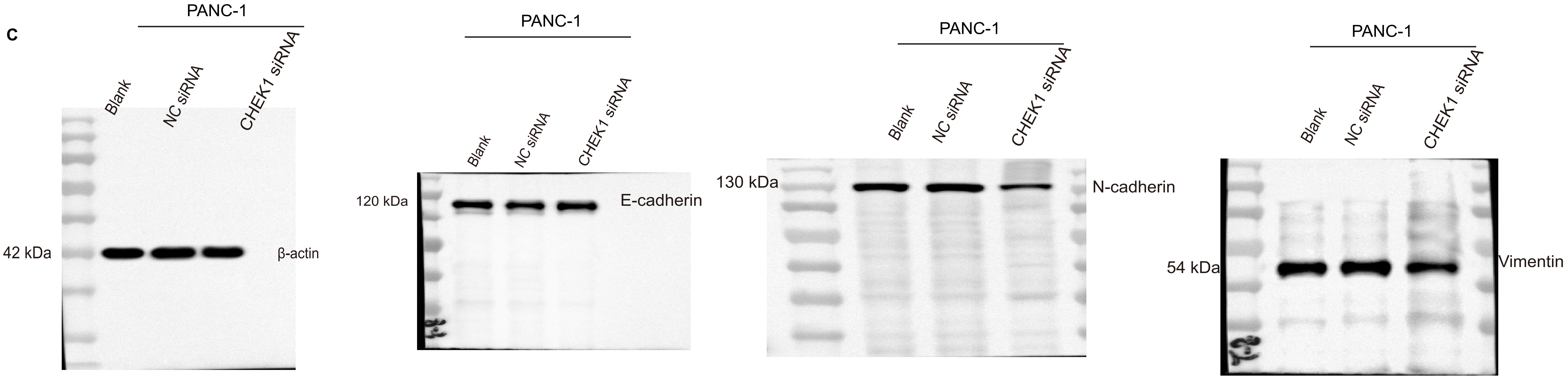

D

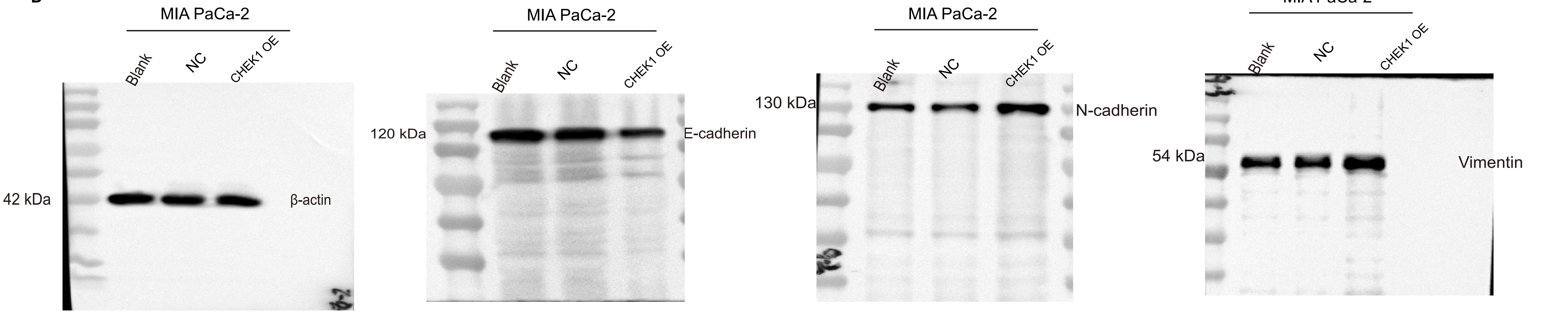

Original WB data

S3 Fig

C

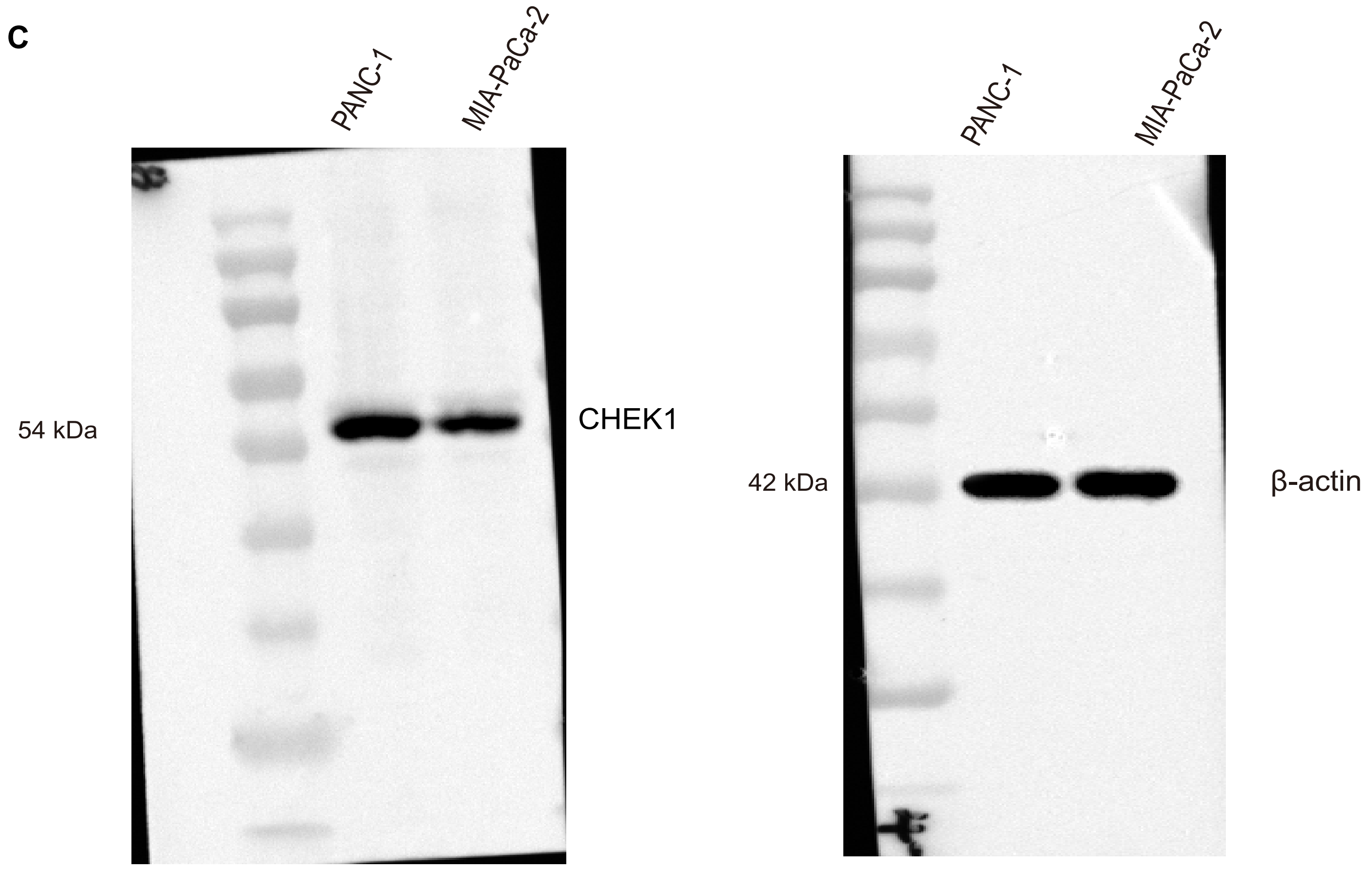

F

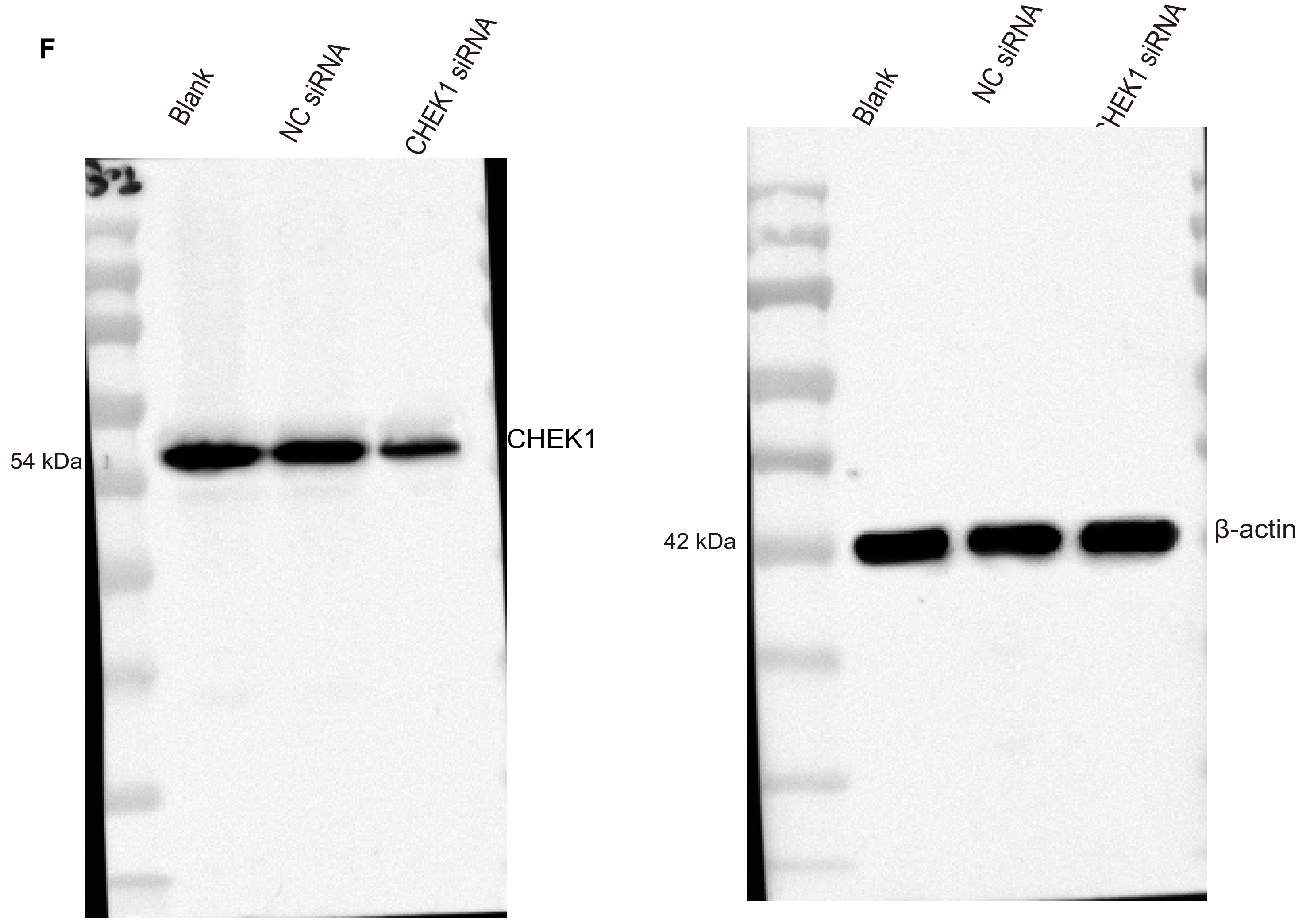

G

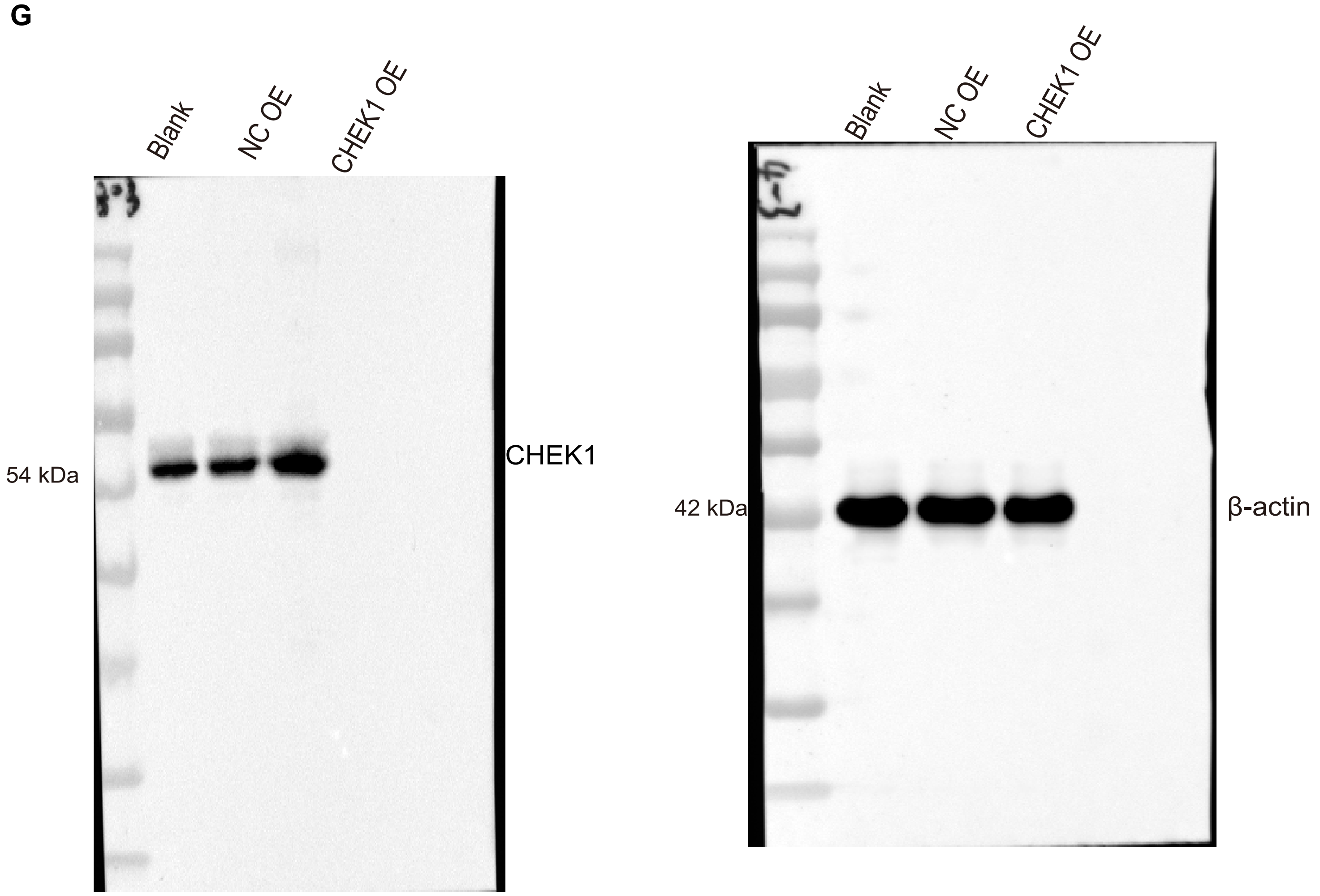

Supplement: S1 data — (PDF) [file pone.0340878.s004.pdf]
