# Supplementary material for: Multi-omics analysis and functional validation of CHEK1 as an independent prognostic biomarker in Pancreatic cancer
Source: PLoS One. 2026 Jan 21;21(1):e0340878. doi: 10.1371/journal.pone.0340878 (PMC12822972; doi:10.1371/journal.pone.0340878)
Supplement: S1 Table — (DOCX) [file pone.0340878.s005.docx]

S1 Table List of primers for qRT-PCR and siRNA

| **Name** |  | **Sequence（5’-3’）** | **Size** |
| --- | --- | --- | --- |
| β-actin | Forward | CATGTACGTTGCTATCCAGGC | 250bp |
|  | Reverse | CTCCTTAATGTCACGCACGAT |  |
| CHEK1 | Forward | GATCAGCTTTTCCCAGCCCA | 108bp |
|  | Reverse | ATTCTTTTGACCAACCGCTGC |  |
| CHEK1-siRNA | Forward | GUGGUUUAUCUGCAUGGUAUU |  |
|  | Reverse | AAUACCAUGCAGAUAAACCAC |  |
| NC-siRNA | Forward | UUCUCCGAACGUGUCACGUTT |  |
|  | Reverse | ACGUGACACGUUCGGAGAATT |  |
