# Supplementary material for: Multi-omics analysis and functional validation of CHEK1 as an independent prognostic biomarker in Pancreatic cancer
Source: PLoS One. 2026 Jan 21;21(1):e0340878. doi: 10.1371/journal.pone.0340878 (PMC12822972; doi:10.1371/journal.pone.0340878)
Supplement: S2 Table — (DOCX) [file pone.0340878.s006.docx]

S2 Table List of antibodies for western blotting

| **Antibody** | **Manufacturer** | **Reference** | **Dilution factor** |
| --- | --- | --- | --- |
| β-actin | Proteintech (Wuhan ,China) | 66009-1-Ig | 1:5000 |
| CHEK1 | Proteintech (Wuhan ,China) | 25887-1-AP | 1:1000 |
| E-cadherin | Proteintech (Wuhan ,China) | 20874-1-AP | 1:5000 |
| N-cadherin | Proteintech (Wuhan ,China) | 22018-1-AP | 1:1000 |
| Vimentin | Proteintech (Wuhan ,China) | 10366-1-AP | 1:1000 |
| Sheep Anti-Rabbit | Jackson (West Grove, PA, USA) | 111-035-003 | 1:10000 |
| Sheep Anti-Mouse | Jackson ( West Grove, PA, USA ) | 111-035-003 | 1:10000 |
