# Supplementary material for: Multi-omics analysis and functional validation of CHEK1 as an independent prognostic biomarker in Pancreatic cancer
Source: PLoS One. 2026 Jan 21;21(1):e0340878. doi: 10.1371/journal.pone.0340878 (PMC12822972; doi:10.1371/journal.pone.0340878)
Supplement: Attachment — Submitted filename: Response to Reviewers.docx [file pone.0340878.s008.docx]

**Dear Editor:**

We very much appreciated the reviewers’ constructive critiques and comments regarding our manuscript, which we have now completely revised according to the reviewers’ suggestions. We have added some additional content to address reviewers' concerns. Many critical changes have been made which are highlighted in red and included in this newly revised version. We offer here our point-by-point response to the reviewers’ questions and concerns:

**Managing Journal Requirements:**

**Q1**: Please ensure that your manuscript meets PLOS ONE's style requirements, including those for file naming. The PLOS ONE style templates can be found at

https://journals.plos.org/plosone/s/file?id=wjVg/PLOSOne_formatting_sample_main_body.pdf and

<https://journals.plos.org/plosone/s/file?id=ba62/PLOSOne_formatting_sample_title_authors_affiliations.pdf>

**A1:** We thank the editor for this reminder. We have carefully revised the manuscript to ensure it fully adheres to PLOS ONE's style requirements, in accordance with the journal's templates.

Q2: Please include your tables as part of your main manuscript and remove the individual files. Please note that supplementary tables (should remain/ be uploaded) as separate "supporting information" files.

**A2:** We are very grateful for the editor's important note. We have revised the manuscript accordingly: （1）The table in the main text (Table 1) should be placed directly within the main manuscript file in an editable format (Word table). （2）Supplementary tables (Table S1 and Table S2) have been uploaded as separate “supplementary information” files.

Q3: Please provide an amended statement that declares *all* the funding or sources of support (whether external or internal to your organization) received during this study, as detailed online in our guide for authors at http://journals.plos.org/plosone/s/submit-now. Please also include the statement “There was no additional external funding received for this study.” in your updated Funding Statement.

Please include your amended Funding Statement within your cover letter. We will change the online submission form on your behalf.

**A3:** Thank you for your guidance. We have amended the Funding Statement as required and included it in our cover letter.

Q4: In the online submission form you indicate that your data is not available for proprietary reasons and have provided a contact point for accessing this data. Please note that your current contact point is a co-author on this manuscript. According to our Data Policy, the contact point must not be an author on the manuscript and must be an institutional contact, ideally not an individual. Please revise your data statement to a non-author institutional point of contact, such as a data access or ethics committee, and send this to us via return email. Please also include contact information for the third party organization, and please include the full citation of where the data can be found.

**A4:**Thank you for raising this issue. We have revised the data availability statement as requested. The contact has been changed from the co-author to the Data Access Committee of the Precision Medicine Institute at Jining Medical University. The institution's contact information can be found in the Data availability statement section of the article.

Q5: PLOS ONE now requires that authors provide the original uncropped and unadjusted images underlying all blot or gel results reported in a submission’s figures or Supporting Information files. This policy and the journal’s other requirements for blot/gel reporting and figure preparation are described in detail at <https://journals.plos.org/plosone/s/figures#loc-blot-and-gel-reporting->

requirements and https://journals.plos.org/plosone/s/figures#loc-preparing-figures-from-image-files. When you submit your revised manuscript, please ensure that your figures adhere fully to these guidelines and provide the original underlying images for all blot or gel data reported in your submission. See the following link for instructions on providing the original image data: https://journals.plos.org/plosone/s/figures#loc-original-images-for-blots-and-gels.

In your cover letter, please note whether your blot/gel image data are in Supporting Information or posted at a public data repository, provide the repository URL if relevant, and provide specific details as to which raw blot/gel images, if any, are not available. Email us at plosone@plos.org if you have any questions.

**A5:** Following the editor’s request，all original, uncropped, and unadjusted imprint images in this document have been uploaded as a Supporting Information file named ‘S1_raw_images’.

Q6: Thank you for stating the following in your manuscript:“This work was supported by the National Natural Science Foundation of China (No. 82002961, 82101867).”We note that you have provided additional information within the Acknowledgements Section that is not currently declared in your Funding Statement. Please note that funding information should not appear in the Acknowledgments section or other areas of your manuscript. We will only publish funding information present in the Funding Statement section of the online submission form.

Please remove any funding-related text from the manuscript and let us know how you would like to update your Funding Statement. Currently, your Funding Statement reads as follows:

“This work was supported by the National Natural Science Foundation of China (No. 82002961, 82101867).” Please include your amended statements within your cover letter; we will change the online submission form on your behalf.

**A6:** Following the editor's advice and in compliance with journal policy requirements, we have removed all references to funding sources from the manuscript and incorporated the revised statement into the cover letter.

Q7: If the reviewer comments include a recommendation to cite specific previously published works, please review and evaluate these publications to determine whether they are relevant and should be cited. There is no requirement to cite these works unless the editor has indicated otherwise.

**A7:** Thanks for your suggestion, we confirm that no specific citations were requested by the reviewers in their comments.

**Reviewer #2:**

Main Comments

Q1: Statistical Analysis, Experimental Design, Data Presentation, and Data Availability

These core areas require significant clarification and improvement. The manuscript would benefit greatly from better structuring of the methodology, clearer data presentation, and proper disclosure of dataset availability.

**A1：**We sincerely thank the reviewers for their critical and constructive comments on the core content of this manuscript. We have thoroughly revised the manuscript to address these issues, significantly enhancing the clarity, rigor, and reproducibility of the research. Key improvements are outlined below:

（1）Statistical Analysis: In response to the reviewer's comment, we have added a new 'Statistical Analysis' subsection to the end of the 'Materials and Methods' chapter. This subsection provides a comprehensive and detailed exposition of statistical methods.and highlighted in red to ensure all changes are clearly visible, line 269-275, page 9-10.

Q2: Figures/Schematic Representations

Consider including the following schematic illustrations to improve clarity and flow:

Figure 1: A diagram summarizing the bioinformatics pipeline, highlighting how data flowed into the functional validation experiments (including methods).

Figure 2: A conceptual model of CHEK1’s proposed role in pancreatic cancer, illustrating its potential as a biomarker. This figure could be included in Discussion.

**A2 (1):** We thank the reviewer for this suggestion. As recommended, we have now added a new diagram as Supplementary Figure 1 (S1 Fig.), which summarizes the bioinformatics pipeline and illustrates how the analysis led to the functional validation experiments.

**A2 (2):** We are grateful to the reviewer for this insightful comment. In response, we have developed a conceptual model that delineates the proposed mechanistic role of CHEK1 in PAAD and its potential clinical implications as a biomarker. This model is now presented as Fig 7 in the Conclusions section.

Minor Comments

Q1: Cell Lines: Clearly explain the biological and molecular differences between MIA-PaCa-2 and PANC-1.

**A1:** Thank you for your valuable suggestions. To clearly and concisely summarize the key differences, we have created a table comparing the fundamental characteristics of the MIA-PaCa-2 and PANC-1 cell lines.See

Table 1: Key Biological and Molecular Characteristics of the MIA-PaCa-2 and PANC-1 Human Pancreatic Cancer Cell Lines. Briefly, the two lines differ notably in their origin (primary pancreas tumor vs. metastatic site), key driver mutations (harboring different KRAS and TP53 alleles), and morphological appearance (epithelioid vs. more fibroblastic). We believe this table will provide readers with a quick and comprehensive reference for the distinct biological and molecular profiles of these commonly used models."

| **Feature** | **MIA-PaCa-2** | **PANC-1** | **Primary Data Sources & Notes** |
| --- | --- | --- | --- |
| **Origin / Pathology** | Pancreatic carcinoma; Poorly differentiated | Pancreatic ductal epithelium; Moderately differentiated | ATCC Database; Deer et al., 2010 |
| **Key Genetic Alterations** |  | | |
| KRAS mutation | Homozygous G12C | Heterozygous G12D | ATCC,COSMIC,CCLE databases |
| TP53 status | Mutated (R248W) | Wild-type | ATCC, COSMIC databases |
| CDKN2A/p16status | Homozygously deleted | Mutated or deleted | Literature consensus |
| **Molecular Subtype** | Basal-like/Quasi-mesenchymal | Classical | Collisson et al., 2011; Moffitt et al., 2015 |
| **Morphology** | attached epithelial with floating rounded cells | epithelial | ATCC Database |
| **Invasive Potential (in vitro)** | Moderate | High | Deer et al., (2010) |

From the perspective of functional tractability, as described in our manuscript (FigS2A), analysis of the CCLE database revealed that PANC-1 and MIA-PaCa-2 represent the two extremes of the CHEK1 expression spectrum. This “High-Low Contrast Model” strategy offers a critical advantage: knocking down CHEK1 in PANC-1 (high expression) allows observation of its loss-of-function phenotype; conversely, overexpressing CHEK1 in MIA-PaCa-2 (low expression) validates its gain-of-function effect. This ensures the robustness and universality of our findings. Second, this functional screening strategy is highly complementary to our previously outlined consideration of molecular heterogeneity. Despite differing baseline CHEK1 expression levels, these cell lines harbor distinct KRAS and TP53 mutation backgrounds. Thus, if CHEK1 demonstrates consistent key functions across such divergent genetic contexts, it significantly enhances the universality and clinical relevance of our findings. In summary, our selection is deliberate: the combination of PANC-1 and MIA-PaCa-2 not only provides an ideal functional comparison model but also enables us to assess the common role of CHEK1 across different molecular subtypes of pancreatic cancer.

**Q2:** Line 85: Provide the web address for Clinical Biosignal House at its first mention.

**A2:** We appreciate the reviewer's suggestions. As requested, we have now provided the official website of Clinical Biosignal House upon its first mention in the paper, See line 86, page 3.

Q3: Line 113: Clarify the comparison groups used for statistical analysis.

**A3:** We appreciate this important comment from the reviewers. We have made comprehensive revisions to the Materials and Methods section where line 113 appears, clearly defining the control group used for analysis. All modifications are highlighted in red in the revised manuscript. See line 126-136, page 5.

Q4: Lines 121-122, 129: These sentences are misplaced. Please move such information to the figure legends where appropriate.

**A4:** We appreciate the reviewers' suggestions. As recommended, the descriptive details in lines 121–122 and line 129 have been moved to the corresponding figure legends, and the Materials and Methods section has been comprehensively revised (see red markings, lines 153–166, page 6).

Q5: Lines 131-132: Rewrite to include the names of the specific R packages used.

**A5:** We thank the reviewer for this suggestion. We have revised the sentence in Lines 131-132 to specify the names and purposes of the key R packages used in our analysis, as requested.See line 161-166, page 6.

Q6: Lines 146-147: Provide at least one reference for the method used. Also clarify that “maestro” and “Seurat” are R libraries, and include the version numbers.

**A6:** We sincerely appreciate the reviewer's comments. However, we regret to inform you that upon verification, we did not obtain valid expression results for the CHEK1 gene in our pancreatic cancer tissue single-cell data. Since this result is not presented in the manuscript, to maintain consistency between the “Materials and Methods” and “Results” sections, we have decided to remove the corresponding analytical method description.

Q7: Lines 146-147 (continued): The t-SNE-based clustering and sub-clustering approach is poorly explained. What was the rationale? How was it implemented? Present and discuss the results accordingly.

**A7:** We appreciate the reviewers' valuable comments. Since no valid expression results for the CHEK1 gene were obtained in this dataset, we have decided to remove the descriptions of analytical methods mentioned by the reviewers—such as t-SNE clustering—from the manuscript to maintain consistency between the “Methods” and “Results” sections.

Q8: Line 207: Indicate the incubation time for the relevant experiments.

**A8:** We appreciate the reviewer's suggestions. The incubation time has been explicitly labeled as “after 12 hours of incubation.” This modification has been highlighted for the reviewer's reference,See line 252-253, page 9.

Q9: Line 218: While the databases used are public, the specific datasets referenced in this study are not accessible. Please provide the repository link or accession numbers.

**A9:**We appreciate the reviewer's important suggestion. All database URLs used in this study have been modified into accessible hyperlinks and are now displayed in blue.

Q10: Lines 232-234: This sentence lacks context and appears disconnected. Please rewrite for clarity.

**A10:** We sincerely appreciate the reviewer's suggestions. We have rewritten the relevant sections of the manuscript as advised to provide a more complete and coherent narrative. See line 290-293, page 10.

Q11: Line 259: Replace “univariable” with “univariate.”

**A11:** We thank the reviewer for this careful observation. The term has been corrected to "univariate" in the result,See line 320, page 11.

Q12: Line 260: Define HR (Hazard Ratio) at first mention.

**A12:** Thank you for this helpful suggestion. As recommended, we have defined "HR" as "Hazard Ratio" upon its first mention in the manuscript.See line 321, page 11.

Q13: Lines 289–291: If “p Spearman” refers to the correlation coefficient, replace p with r. To avoid redundancy, report both r and p-values in Figure 3, and simplify the text to state that CHEK1 correlates significantly with tumor proliferation, G2M checkpoint, DNA replication, and DNA repair. Also, add “Score” to the y-axis labels.

**A13:** We sincerely thank the reviewer for this valuable feedback. We have carefully revised the manuscript according to the suggestions, We have revised the manuscript by standardizing the correlation coefficient notation to "r", annotating Figure 3 with both r and p-values, simplifying redundant textual descriptions (See line 357-362, page 12-13), and clarifying y-axis labels with "Score" as suggested.

Q14: siRNA Experiments: Please explain why siRNA treatment leads to reduced colony formation. Is this a direct effect on proliferation or another mechanism?

**A14:** We thank the reviewer for highlighting the important issue of reduced colony formation after CHEK1 knockdown. Based on our experimental data, siRNA-mediated knockdown of CHEK1 reduces clonogenicity through a process that directly affects cell proliferation. The core mechanism is as follows: CHEK1 deficiency induces G1 phase arrest, thereby reducing the proportion of cells in S phase. This directly inhibits DNA replication and cell division, ultimately weakening the cells' long-term proliferative capacity and clonogenic potential (Fig4A and 4C; Fig 5A).

Q15: Line 318: Define EMT (epithelial-mesenchymal transition) at first mention.

**A15:** We thank the reviewer for this suggestion. The abbreviation "EMT" has now been defined as "epithelial-mesenchymal transition" at its first mention in the manuscript as recommended.See line 402-403 page 14.

Q16: Lines 345–346: The claim that multivariate analysis supports CHEK1 as an independent prognostic factor is vague. Please expand this section and include relevant literature references.

**A16:** We sincerely appreciate the reviewer's insightful suggestion. We have comprehensively revised the Discussion section (line 345-346) to provide a more thorough explanation of why multivariate analysis supports CHEK1 as an independent prognostic factor, see line 431-442,page 15. Based on the reviewers' suggestions, we have also provided supporting literature,see line 442-446,page 15.

Q17: Statistical Testing: Indicate the normality test used for each dataset. Justify the use of Pearson vs. Spearman correlation analyses.

**A17:** Thank you for your valuable feedback on this manuscript and for your attention to our data analysis methods. We have provided a detailed explanation of the statistical test selection based on your suggestions and have revised the manuscript accordingly. Please refer to the Materials and Methods section and the Statistical Analysis section.

In this study, the selection of correlation analysis methods is based on the distribution characteristics of variables and the nature of their relationships. For variables that follow a normal distribution and exhibit linear relationships, Pearson correlation analysis is employed to precisely quantify the strength and direction of their linear association. For variables that do not follow a normal distribution, contain outliers, or demonstrate monotonic nonlinear relationships, Spearman's rank correlation analysis is utilized.

Q18: Cell Line Coverage: If siRNA and overexpression experiments were conducted in both PANC-1 and MIA-PaCa-2 cells, then all associated results (colony formation, cell cycle analysis, and migration assays) must be presented for both lines.

**A18:** We thank the reviewer for this critical comment regarding the presentation of data from both cell lines. Our selection of PANC-1 and MIAPaCa-2 was not arbitrary but was strategically based on our preliminary bioinformatic analysis of the CCLE database, which identified them as representative models with the highest (PANC-1) and lowest (MIAPaCa-2) endogenous expression levels of CHEK1 among pancreatic cancer cell lines (as shown in our original Figure S2A). This deliberate choice allowed us to test our central hypothesis under two contrasting biological contexts: In PANC-1 (high CHEK1 background), we aimed to investigate whether knocking down CHEK1 would disrupt a potentially "addicted" pathway. In MIAPaCa-2 (low CHEK1 background), we sought to determine whether overexpressing CHEK1 could enhance oncogenic phenotypes, and conversely, whether knocking it down would still have an effect-a scenario that would strongly argue for a specific and fundamental role of CHEK1 beyond merely reducing high baseline levels.

Q19: Figure 1:

(1) Add data points to all boxplots.

**A19 (1):** We appreciate the reviewer's important suggestion to enhance data transparency by adding individual data points to box plots. We fully agree with this principle. Accordingly, we have revised Fig 1A and Supplementary Fig S1B to include all data points. Regarding the other subplots in Fig 1, these specific figures were generated directly by an online analysis platform that does not support overlaying data points and does not provide access to the underlying raw data. Therefore, it is unfortunately not possible to replace them with figures showing individual data points.

(2): Replace asterisks with exact p-values.

**A19 (2):** We appreciate the valuable comments provided by the reviewers. Accordingly, we have replaced the asterisks in Fig 1 with precise p-values to enhance the accuracy and clarity of the results.

(3): Justify the use of z-values in Figure 1B, or standardize the y-axis across subpanels.

**A19 (3):** Thanks for the reviewer’s comments. In the Fig.1B, Z-values represent standard deviations from the median across samples for the given cancer type. Log2 Spectral count ratio values from Clinical Proteomic Tumor Analysis Consortium (CPTAC) were first normalized within each sample profile, then normalized across samples.

**Q20:** Figure 2B : Add a legend or label to the x-axis.

**A20:** We thank the reviewer for pointing this out. The x-axis of Figure 2B has now been labeled as "Time in days " to clarify the timeline of the experiment, as recommended.

**Q21：**Figure 5: Replace barplots with boxplots including data points.

**A21:**We thank the reviewer for this suggestion. As recommended, we have replaced the bar plots in Figure 5 with box plots that include all individual data points. The revised figure now provides a more detailed representation of the data distribution. Please see the updated Fig 5

**Q22：**Figure 6: Add p-values directly onto plots instead of using asterisks.

Replace barplots with boxplots showing individual data points.

**A22:** We thank the reviewer for these suggestions. We have revised Fig 6 accordingly: All p-values are now directly annotated on the plots. All bar plots have been replaced with box plots that show individual data points.

**Q23：**Supplementary File S1: Maintain consistent plotting style throughout. I recommend using boxplots with individual data points for all relevant figures.

**A23:** We thank the reviewer for the suggestion to maintain a consistent plotting style. We have revised the Supplementary File S1 accordingly: We noticed that panel S1B and Figure 1A were derived from the same database. To avoid redundancy, we have removed Supplementary Figure S1B. As recommended, we have unified the plotting style by replacing the violin plot in the original panel S1C (now labeled S1B) with a box plot showing individual data points. This box plot was directly sourced from the TNMplot website, which provides both violin and box plot representations for the same data. This change ensures consistency with the plotting style of the other figures. We believe these changes have improved the consistency and clarity of the supplementary information.

**Reviewer #3**

**Q1：**In Fig. 1A, only 4 control samples were included. While the authors state that the result is significant, it is not clear what statistical test was used. To this point, asterisks are included in this and some other figures without explanation.

**A1:** We thank the reviewer for this valuable comment. To address the concerns regarding the small control sample size (n=4) and the unspecified statistical test in Fig. 1A, we have significantly expanded the control group to 332 samples by incorporating 328 normal samples from the GTEx database. Furthermore, we have explicitly stated that the statistical significance was determined using a T-test and have now clearly labeled the P-values in Fig. 1 and related figures as suggested. We believe these revisions have substantially strengthened the statistical power and clarity of our results.

**Q2:** It is curious that the colony formation of PANC-1 and MIA-PaCa-2 cells is nearly identical (~16%, Figs 4 A & B, blank columns) given that the authors chose these two cell lines because of their differences in CHEK1 expression (Fig. S2). It would be useful for experiments to include siRNA and overexpression in both cell lines. Furthermore, a “normal” (non-cancer) pancreatic cell line should be tested to determine if the effects seen are cancer specific.

**A2:** We appreciate the reviewer's insightful comment. We agree that despite differing levels of CHEK1 expression, PANC-1 and MIA-PaCa-2 cells exhibit similar baseline colony-forming capacity—a fascinating phenomenon that underscores the complexity of cancer signaling. Our core finding is not based solely on expression levels, but rather on the starkly different responses observed after CHEK1 inhibition (Figure 4A and B), revealing their differential functional dependence on CHEK1. We will revise the Discussion section to reframe this phenomenon: rather than a contradiction, it underscores the redundancy of signaling pathways in PDAC and the context-dependent nature of CHEK1 function. We acknowledge the value of comparing with normal cells and identify this as a key direction for future research.

**Q3:** As the authors point out in their introduction, p53 status is an important factor in CHEK1 function. The two cell lines employed both have TP53 mutations. The authors have mined data on many pancreatic cancer cell lines (Fig. S2) and should include more cell lines in their various experimental procedures.

**A3:** We thank the reviewer for this insightful comment regarding p53 status. Upon verification via ATCC and COSMIC databases, we must clarify a critical point: PANC-1 is TP53 wild-type, while MIA-PaCa-2 is TP53 mutated (R248W). This fact, which we apologize for omitting initially, actually reveals the strategic rationale behind our cell line selection. Our models constitute an ideal paired system to investigate the functional interplay between p53 status and CHEK1, as we have concurrently identified an inverse relationship where PANC-1 (TP53-WT) exhibits high CHEK1 expression that promotes proliferation, whereas MIA-PaCa-2 (TP53-Mut) exhibits low CHEK1 expression. Therefore, the core of our study is not merely profiling multiple lines, but rather conducting a deep mechanistic analysis of this informative contrast. The differential responses we report are central to understanding how p53 context shapes CHEK1 dependency. We will revise the manuscript to forefront this rationale and discuss how our findings using this specific model provide novel insights into this interaction, beyond what a broader but more superficial screen could achieve.

**Q4:** Although much of the manuscript is nicely written, there are several instances where editing is needed. For example, there are incomplete sentences and in one case, a paragraph that seems out of place (paragraph beginning on line 285). Thorough editing and proof-reading are required.

**A4:** Thank you for your valuable feedback on the manuscript's structure and language. We have meticulously edited and proofread the entire text based on your suggestions, correcting incomplete sentences and grammatical errors. Regarding the logical issue you specifically pointed out in the paragraph at line 285, we have relocated it to lines 294–300 , page 10-11 and revised and polished the content to enhance its coherence with the surrounding context. All modifications are highlighted in red within the manuscript for your review. We believe these revisions have significantly improved the readability and logical flow of the manuscript.

.
