# Supplementary material for: Multi-omics analysis and functional validation of CHEK1 as an independent prognostic biomarker in Pancreatic cancer
Source: PLoS One. 2026 Jan 21;21(1):e0340878. doi: 10.1371/journal.pone.0340878 (PMC12822972; doi:10.1371/journal.pone.0340878)
Supplement: Attachment — Submitted filename: Response_to_Reviewers_auresp_2.docx [file pone.0340878.s009.docx]

**Dear Editor,**

We sincerely appreciate the thoughtful and constructive feedback from the reviewers on our manuscript. We have carefully revised the manuscript in accordance with all of the reviewers’ suggestions, and have incorporated additional content to address their concerns. Key revisions are highlighted in red within the newly revised version. Below, we provide a point-by-point response to each of the reviewers’ questions and comments.

**Journal Requirements:**

Q1: If the reviewer comments include a recommendation to cite specific previously published works, please review and evaluate these publications to determine whether they are relevant and should be cited. There is no requirement to cite these works unless the editor has indicated otherwise.

Please review your reference list to ensure that it is complete and correct. If you have cited papers that have been retracted, please include the rationale for doing so in the manuscript text, or remove these references and replace them with relevant current references. Any changes to the reference list should be mentioned in the rebuttal letter that accompanies your revised manuscript. If you need to cite a retracted article, indicate the article’s retracted status in the References list and also include a citation and full reference for the retraction notice.

A1: In response to the journal's requirements, we confirm that all necessary checks have been completed. Notably, the reviewers did not suggest any changes to the references. We have nevertheless thoroughly verified the reference list for completeness, accuracy, and proper formatting, and confirmed that no retracted articles are cited. All relevant updates are reflected in the revised manuscript.

**Reviewer #2:**

**Q1**: The manuscript has been significantly improved. However, some minor issues remain to be addressed. For instance, the authors indicate in their Response to Reviewers that Table 1 contains the “Key biological and molecular characteristics of the MIA-PaCa-2 and PANC-1 human pancreatic cancer cell lines,” but in the revised manuscript Table 1 is titled “Clinical characteristics of CHEK1 in pancreatic cancer samples.” Please provide the correct table.

**A1:** We thank the reviewer for carefully pointing out the confusion regarding the table numbering. We sincerely apologize, as this likely resulted from insufficient clarity in our description within the Response to Reviewers.

The "Table 1" referenced in our response letter is a supplementary table specifically prepared to address your earlier comment regarding "clarifying the biological and molecular differences between the two cell lines." Its content details the "Key biological and molecular characteristics of the MIA-PaCa-2 and PANC-1 human pancreatic cancer cell lines." This table was intended solely to directly respond to your inquiry and was not planned for inclusion in the main manuscript text.Change "See Table 1" to "Please see the table below."

The "Table 1" in the main manuscript is the pre-existing table from the original submission that analyzes clinical data, titled "Clinical characteristics of CHEK1 in pancreatic cancer samples."

Therefore, the response to the first issue raised in the reviewer's minor comments has been revised.

**Minor Comments:**

**Q1:** Cell Lines: Clearly explain the biological and molecular differences between MIA-PaCa-2 and PANC-1.

**A1:** Thank you for your valuable suggestions. To clearly and concisely summarize the key differences, we have created a table comparing the fundamental characteristics of the MIA-PaCa-2 and PANC-1 cell lines. Please see the table below: Key Biological and Molecular Characteristics of the MIA-PaCa-2 and PANC-1 Human Pancreatic Cancer Cell Lines. Briefly, the two lines differ notably in their origin (primary pancreas tumor vs. metastatic site), key driver mutations (harboring different KRAS and TP53 alleles), and morphological appearance (epithelioid vs. more fibroblastic). We believe this table will provide readers with a quick and comprehensive reference for the distinct biological and molecular profiles of these commonly used models."

| **Feature** | **MIA-PaCa-2** | **PANC-1** | **Primary Data Sources & Notes** |
| --- | --- | --- | --- |
| **Origin / Pathology** | Pancreatic carcinoma; Poorly differentiated | Pancreatic ductal epithelium; Moderately differentiated | ATCC Database; Deer et al., 2010 |
| **Key Genetic Alterations** |  | | |
| KRAS mutation | Homozygous G12C | Heterozygous G12D | ATCC,COSMIC,CCLE databases |
| TP53 status | Mutated (R248W) | Wild-type | ATCC, COSMIC databases |
| CDKN2A/p16status | Homozygously deleted | Mutated or deleted | Literature consensus |
| **Molecular Subtype** | Basal-like/Quasi-mesenchymal | Classical | Collisson et al., 2011; Moffitt et al., 2015 |
| **Morphology** | attached epithelial with floating rounded cells | epithelial | ATCC Database |
| **Invasive Potential (in vitro)** | Moderate | High | Deer et al., (2010) |

From the perspective of functional tractability, as described in our manuscript (FigS2A), analysis of the CCLE database revealed that PANC-1 and MIA-PaCa-2 represent the two extremes of the CHEK1 expression spectrum. This “High-Low Contrast Model” strategy offers a critical advantage: knocking down CHEK1 in PANC-1 (high expression) allows observation of its loss-of-function phenotype; conversely, overexpressing CHEK1 in MIA-PaCa-2 (low expression) validates its gain-of-function effect. This ensures the robustness and universality of our findings. Second, this functional screening strategy is highly complementary to our previously outlined consideration of molecular heterogeneity. Despite differing baseline CHEK1 expression levels, these cell lines harbor distinct KRAS and TP53 mutation backgrounds. Thus, if CHEK1 demonstrates consistent key functions across such divergent genetic contexts, it significantly enhances the universality and clinical relevance of our findings. In summary, our selection is deliberate: the combination of PANC-1 and MIA-PaCa-2 not only provides an ideal functional comparison model but also enables us to assess the common role of CHEK1 across different molecular subtypes of pancreatic cancer.

**Q2:** I appreciate that web links have been provided; however, in the interest of transparency, I would like the datasets used for the statistical analyses (such as those described in lines 114–122) to be made available as supplementary material or uploaded to a public repository.

**A2:** We thank you for your suggestion and sincerely apologize for any misunderstanding caused by the lack of clarity in our previous description.In response to your comments, we have revised the content in lines 114–122 to clarify that "all analyses were conducted using the standardized modules of the specified online website."See red line 125-138, page 5. Additionally, the correlation analysis of the CHEK1 gene and its associated pathways in the Materials and Methods section has been revised. See red lines 150–153 on page 5 and red lines 161–162 on page 6.

The public data underwent normalization processing on the platform, generating formatted data ready for direct analysis. All data used in this study can be accessed through the fixed analysis modules of the website, as described in the manuscript.To meet the reviewers' requirements while preserving the article's structure, we have downloaded the raw data used for analysis on the platform for the reviewers' review. This data is provided in separate files named “Raw Data for Pathway Correlation Analysis” and “Raw Data for Prognostic Model Analysis.”

**Q3：**Please provide the N in the legend of those plots where datapoints were not depicted.

**A3:** We appreciate the valuable suggestions provided by the reviewers. In accordance with their requests, we have thoroughly reviewed all figures that do not directly display data points and confirmed that only Figures 1C and 1D lack explicit labeling of data points. Regarding the suggestion for supplemental sample size information, we have cross-referenced the original data sources and relevant literature and confirmed that the currently cited data does not provide specific sample size details. We have also actively sought alternative publicly available data platforms to meet the reviewers' requirements but have not yet identified a suitable data source that simultaneously covers both Stage and Grade analyses. The relevant references are listed below for the reviewers' further consideration.

**References: Ru B, Wong CN, Tong Y, Zhong JY, Zhong SSW, Wu WC, Chu KC, Wong CY, Lau CY, Chen I, Chan NW, Zhang J. TISIDB: an integrated repository portal for tumor-immune system interactions. Bioinformatics. 2019 Oct 15;35(20):4200-4202. doi: 10.1093/bioinformatics/btz210. PMID: 30903160.**

**Q4:** Finally, the manuscript still contains multiple spacing and formatting errors in the text; please revise it thoroughly.

**A4:** Thank you for this important reminder. We sincerely apologize for the spacing and formatting errors that remained in the previous version. We have now conducted a thorough, line-by-line revision of the entire manuscript to correct these issues and have also polished the language. Please refer to the 'Revised Manuscript with Track Changes' document for details.

Reviewer #3:

**Q1:** The authors have addressed the original concerns, especially those regarding statistical tests and descriptions. A couple of recommended additional experiments were not conducted, but solid rationales for not pursuing them were provided. The issue that still remains is the quality of the English. There are many errors, too many to list here. It is recommended that the authors seek independent editorial help.

**A1:** We sincerely thank the reviewer for their valuable feedback. In accordance with the suggestions, the entire manuscript has undergone language polishing and revision, with all changes highlighted in red. Please refer to the 'Revised Manuscript with Track Changes' document for details.
